# Toward a Generic Framework for Mission Planning and Execution with a Heterogeneous Multi-Robot System

**DOI:** 10.3390/s24216881

**Published:** 2024-10-26

**Authors:** Mohsen Denguir, Ameur Touir, Achraf Gazdar, Safwan Qasem

**Affiliations:** 1 Department of Software Engineering, College of Computer and Information Sciences, King Saud University, Riyadh 11543, Saudi Arabia; denguir@ksu.edu.sa; 2 Department of Computer Science, College of Computer and Information Sciences, King Saud University, Riyadh 11543, Saudi Arabia; touir@ksu.edu.sa; 3 Department of Computer Engineering, College of Engineering, Al Yamamah University, Riyadh 11512, Saudi Arabia; s_qasem@yu.edu.sa

**Keywords:** heterogeneous multi-robot systems, UGV, UAV, mission planning, decentralized control, formation control, formation stability, task allocation

## Abstract

This paper presents a comprehensive framework for mission planning and execution with a heterogeneous multi-robot system, specifically designed to coordinate unmanned ground vehicles (UGVs) and unmanned aerial vehicles (UAVs) in dynamic and unstructured environments. The proposed architecture evaluates the mission requirements, allocates tasks, and optimizes resource usage based on the capabilities of the available robots. It then executes the mission utilizing a decentralized control strategy that enables the robots to adapt to environmental changes and maintain formation stability in both 2D and 3D spaces. The framework’s architecture supports loose coupling between its components, enhancing system scalability and maintainability. Key features include a robust task allocation algorithm, and a dynamic formation control mechanism, using a ROS 2 communication protocol that ensures reliable information exchange among robots. The effectiveness of this framework is demonstrated through a case study involving coordinated exploration and data collection tasks, showcasing its ability to manage missions while optimizing robot collaboration. This work advances the field of heterogeneous robotic systems by providing a scalable and adaptable solution for multi-robot coordination in challenging environments.

## 1. Introduction

In modern robotics, executing complex tasks often necessitates the collaboration of multiple robots. To achieve a common objective, these robots must work in tandem, coordinating their actions to accomplish various lower-level tasks. Complex missions such as foraging, exploration, and flocking require not only cooperative behavior but also the ability to form and maintain specific patterns. For successful mission execution, a robust mission planning and task allocation system is essential, enabling robots to synchronize their efforts toward a shared goal.

Unmanned ground vehicles (UGVs) and unmanned aerial vehicles (UAVs) each bring unique strengths and limitations to these missions. UGVs, while highly effective in ground operations, often face challenges due to their limited sensing range. In contrast, UAVs excel in providing extensive aerial surveillance and reconnaissance, although they typically lack the payload capacity required for certain tasks. The complementary nature of UGVs and UAVs suggests that integrating them into a heterogeneous multi-robot system can leverage the strengths of both, offering a more versatile and capable solution than homogeneous systems [1,2].

This paper introduces a generic framework designed to facilitate mission planning and execution within a heterogeneous system comprising UGVs and UAVs. The framework emphasizes maintaining both 2D and 3D formations, addressing two primary challenges: formation control and stability.

Maintaining the stability of multi-robot formations, particularly in heterogeneous systems, relies heavily on robust communication protocols. These protocols ensure real-time information exchange, allowing the robots to make dynamic position adjustments based on both external environmental changes and internal mission demands.

In the proposed framework, we leverage ROS 2 communication technology to enable real-time information exchange between the UAVs and UGVs involved in the formation. ROS 2 provides a scalable, reliable, and decentralized communication infrastructure that supports the dynamic, distributed nature of multi-robot systems. This ensures that the robots can make rapid, coordinated position adjustments in response to both environmental changes and evolving mission requirements. Effective communication is the backbone of coordinated actions within these formations, as it enables the system to remain cohesive and responsive during complex operations.

However, communication alone is not sufficient for managing the intricacies of multi-robot missions. To fully realize the potential of heterogeneous systems, additional components—such as control algorithms, task allocation strategies, and system-wide adaptability—must be seamlessly integrated. The proposed framework in this paper addresses these challenges by providing a structured approach to mission planning and execution. It is designed to enhance the efficiency of multi-robot systems through not only reliable communication but also the integration of essential functional components, which we propose: drivers, controllers, workers, aggregators, and oracles. These components are the primary building blocks used to handle the complexity of mission scenarios. As detailed later, this framework simplifies the initialization and coordination of these blocks, ensuring smoother execution across a variety of mission environments.

Thus, the main contributions of this paper are as follows:

- Designing a framework that facilitates hybrid multi-robot mission planning and execution. The framework is intended to be integrated, in the future, into software that allows a user (by means of a graphical wizard) to go through all the steps required to plan and execute a mission involving UGVs and UAVs.
- Illustrating through a simple simulated mission deploying hybrid robots, how the building blocks of the framework (i.e., drivers, controllers, oracles) could be implemented and integrated altogether to accomplish the illustrative mission presented as a proof of concept. The complex mission will rather necessitate more instances of our framework building blocks with a higher customization level of the written code to fulfill the mission.

Recent studies have highlighted the benefits of integrating UGVs and UAVs into a cohesive system, demonstrating improved coverage and task efficiency in various scenarios, such as search and rescue operations and environmental monitoring [1,2]. Moreover, advancements in decentralized planning and control strategies for UAV-UGV teams have further optimized these systems, making them more resilient and adaptable to complex environments [3].

The subsequent sections of this document are structured in the following manner. In Section 2, we provide an overview of the primary literature pertaining to the topic. The Section 3 is specifically devoted to providing a detailed explanation of the suggested framework. The Section 4 is a case study of a straightforward mission that demonstrates the application of the framework. In Section 5, we provide a concise summary of the primary contribution and outline potential future research directions.

## 2. Related Work

The stability of multi-robot formations, especially in 3D environments, is essential for ensuring smooth coordination and functionality during complex tasks. To maintain cohesive movement, robots must dynamically adjust to environmental changes and obstacles. Recent advancements have introduced novel algorithms for formation control, enabling these systems to operate more robustly and efficiently, even in challenging conditions. This section investigates some key challenges and issues associated with multi-robot formations, including formation stability, control, coordination, communication, task allocation, scalability, and robustness.

### 2.1. Formation Stability and Control in 3D Space

The 3D formation control is a crucial aspect in applications such as autonomous aerial, ground, and underwater vehicles. Research on control strategies has increasingly focused on developing decentralized approaches to improve flexibility and robustness. Wei et al. [4] proposed a distributed consensus-based approach for multi-robot formation control in 3D environments, allowing for dynamic adaptability when robots encounter uncertain factors or failures. Similarly, Yan et al. [5] demonstrated how formations in UAV swarms can be efficiently controlled using model predictive control (MPC) techniques, ensuring precise coordination even in an environment with external disturbances.

Various studies highlighted the trade-offs between decentralized flexibility and centralized efficiency, offering hybrid solutions that can be applied to diverse robotic tasks such as exploration, path planning, and coordination. Dai et al. [6] proposed a hybrid decentralized and centralized training and execution strategy for path planning in multi-robot systems. This approach initially decentralizes path planning using a deep reinforcement learning algorithm, where each robot independently plans its path. Centralized control is then used to detect potential collisions and optimize overall system coordination before execution, balancing computational efficiency and system performance.

Li et al. [7] introduced a hybrid approach that combines leader–follower and virtual structure methods. This approach improves the stability of formations by allowing flexibility in the relative positions of the robot while treating the formation as a cohesive whole. The leader defines the path, and followers adjust accordingly, while the virtual structure maintains a rigid geometrical relationship within the group, creating a balance between adaptability and structural integrity. This hybrid method is significant in environments where terrain or obstacles may suddenly change, requiring real-time adjustments without compromising overall formation stability.

### 2.2. Heterogeneity in Multi-Robot Systems: Opportunities and Challenges

Heterogeneous multi-robot systems, comprising robots with diverse capabilities, offer significant advantages for complex tasks requiring specialization. However, heterogeneity also brings challenges in terms of coordination, task allocation, and communication. Coordination in heterogeneous multi-robot systems has advanced significantly, particularly with the integration of machine learning techniques. Deep reinforcement learning (DRL) has gained prominence in enabling heterogeneous robots to autonomously learn and execute cooperative behaviors. Brotee et al. [8] applied two DRL algorithms to train a mixed team of UAVs and UGVs, demonstrating that this approach allows the system to improve efficiency in path planning in an obstructed environment in terms of target navigation time and task completion rate. The key advantage of DRL lies in its adaptability—robots can learn optimal strategies from experience and interactions with the environment, significantly reducing the need for manual reprogramming.

Further advancements, such as those by Zhang et al. [9], have focused on integrating DRL with MPC to achieve collision-free navigation for mobile robots while improving computational efficiency. Moreover, Wang et al. [10] explored the use of transfer learning in DRL to improve task adaptation across heterogeneous robots. They proposed a method where a knowledge transfer mechanism allows information learned by one type of robot to be applied to another. This approach enhances learning efficiency and enables the system to adapt to new tasks more rapidly.

### 2.3. Task Allocation in Heterogeneous Systems

Task allocation remains a critical challenge in heterogeneous systems. Efficient task distribution must account for the robots’ varying capabilities and the dynamic nature of tasks. Liu et al. [11] introduced a dynamic task allocation framework that handles large-scale multi-robot systems. Their method leverages distributed algorithms to dynamically partition and distribute tasks among heterogeneous robots, ensuring effective collaboration and scalability. This approach is particularly suited to environments where task requirements and robot capabilities vary. Notomista et al. [12] proposed an optimal task allocation strategy for heterogeneous multi-robot systems. Their approach focuses on allowing robots with different capabilities to collaborate efficiently by solving an optimization problem that prioritizes tasks based on the specific abilities of each robot. The algorithm ensures that tasks are distributed optimally among the robots while minimizing energy consumption and maintaining system efficiency.

Gianni et al. [13] proposed a framework for role and task allocation in cooperative heterogeneous multi-robot systems. Tasks are modeled using multidimensional relational structures that define collaborative tasks through temporal and spatial relationships. The framework incorporates learning mechanisms and tensor-based geometrical reasoning for collaborative tasks.

Lei et al. [14] proposed a framework of team-based multi-robot task allocation and path planning for robot exploration missions through a convex optimization-based distance optimal model. They developed a distance model to minimize the traveled distance between robots and their goals. The framework fuses task decomposition, allocation, and path planning. Elfakharany et al. [15] presented a DRL-based method that is used to perform multi-robot task allocation and navigation in an end-to-end fashion. They use a policy that processes in a decentralized manner, mapping raw sensor measurements to the steering commands of the robot. They claim that their model works without the need to construct a map of the environment. They also provided a metric called the Task Allocation Index, which measures the performance of the process of task allocation and navigation.

Other researchers, such as Wen et al. [16], have explored evolutionary algorithms for adaptive task allocation, particularly in large-scale heterogeneous systems. The proposed techniques are adaptive and scalable, enabling robots to autonomously adjust their roles based on environmental changes or team member failures.

### 2.4. Scalability and Robustness in Large-Scale Systems

As multi-robot systems grow in size and complexity, scalability and robustness become crucial considerations. Wang et al. [17] implemented an independent learning and parameter-sharing approach, allowing single-agent reinforcement learning algorithms to be expanded to multi-agent contexts. They stated that the proposed approach solves their problem of scalability. Regarding the observation issues, they proposed an oracle-guided two-stage training and execution method using the flock center in the training stage while eliminating reliance on it during execution. They produced the oracle-guided observations and developed a simulation environment for experimental purposes. Jiang et al. [18] proposed a decentralized cooperative control method for multi-robot formations using deep neural networks, in which inter-robot communication is modeled by a graph neural network. They use LiDAR sensor data as input. A control policy is learned from demonstrations that are provided by a specific controller for decentralized formation control. They affirm that even though their proposed solution is trained with a fixed number of robots, the learned control policy is scalable.

Also, Blockchain technology has emerged as a promising solution for ensuring secure coordination and data integrity in large-scale systems. Strobel et al. [19] compared the blockchain approach to existing consensus protocols in the context of reaching consensus in robot swarms in the presence of Byzantine robots. They concluded that blockchain-based consensus methods give better results but suffer from delays, which makes them unsuitable if the system needs quick responses. Xiong et al. [20] propose a secure collaborative computing framework based on blockchain technology. They designed a lightweight blockchain scheme with reduced computational and storage requirements. This scheme was used for UAV ad hoc Networks, to reduce consensus overhead and establish trust relationships among UAVs. Blockchain provides a decentralized, tamper-proof method for recording transactions and coordinating actions, ensuring that even in the presence of network failures or communication delays, robots can maintain accurate records of their activities.

## 3. The Generic Framework for Mission Planning and Execution

**Figure 1:**
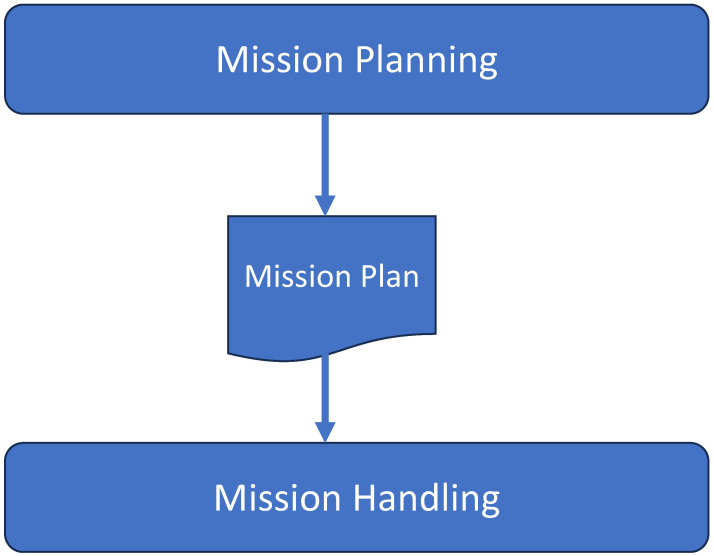
The two main parts of the proposed framework include the conceptual level.

The proposed framework consists of two primary components. A mission planner is tasked with (i) creating a mission, (ii) developing a plan based on available resources, and (iii) assessing its feasibility. By resources, we refer to robots (UGV/UAV) and their capabilities (camera, laser, payload, etc.). A mission handler focuses on ensuring and orchestrating the proper execution of the plan that the mission planner has provided. Figure 1 shows the framework components.

As depicted in Figure 1, the framework is divided mainly into two parts. The first part processes and validates the mission plan, whereas the second part is responsible for controlling and orchestrating the different actors to ensure the achievement of the planned mission.

The two components of the framework are loosely coupled. This enables better maintainability, flexibility, and reusability of the components of the framework. This will allow us to easily implement the necessary software modules required by a multi-robot mission execution by means of instantiating the different components of the framework. A detailed explanation of these components is given in the current section.

### 3.1. Mission Planning

Within the realm of 3D formation involving the execution of missions by multiple robots, the mission planner (MPL) functions as a software module is tasked with evaluating the proposed mission, creating a plan for its execution, checking its feasibility based on available resources (robot types and their tier capabilities), and allocating various mission tasks to these resources.

The mission planner plays a crucial role in orchestrating the deployment of the involved robots to accomplish a set of complex tasks efficiently. To attain this goal, the MPL uses a set of criteria to ensure the success of the mission in particular: (i) The MPL reviews the mission objectives and requirements to assess what needs to be done. (ii) It checks the feasibility of the mission within the available robots, considering their types and capabilities. (iii) It assigns tasks to the most suitable robots, matching their skills with the mission’s needs, while optimizing the resource by balancing the workload and ensuring efficient task execution.

**Figure 2:**
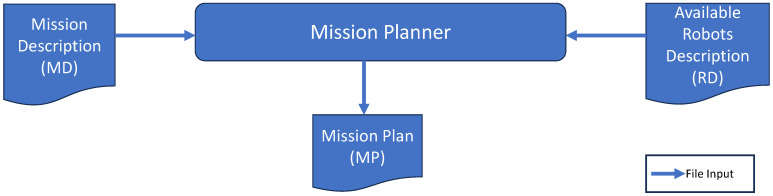
The inputs of the mission planner module.

As depicted in Figure 2, the mission planner needs to receive a mission description as well as a description of the available robots. The mission description defines the different aspects of the mission, including tasks, subtasks, their dependencies, and the required capabilities to execute each one of them. In the sequel, we define each of these concepts. On the other hand, the robot description expresses the specifications and capabilities of the available robots. Figure A2 and Figure A3 are examples of the mission and robot descriptions.

#### 3.1.1. Tasks and Sub-Tasks Definition

A task can be broken down into elementary tasks that will be called sub-tasks. An elementary task cannot be further broken down.

Let *T* denote the set of tasks, *E* denote the set of elementary tasks, and *C* denote the set of robot capabilities.

We define functions start and end, which associate tasks with their start and end times, respectively.

We define a function sub that associates each task with all the sub-tasks that compose it:

$$\begin{array}{cc} sub: & T\to \;SE\;\mathrm{where}\;SE\;\mathrm{is}\;\mathrm{the}\;\mathrm{set}\;\mathrm{sequences}\;\mathrm{of}\;\mathrm{elementary}\;\mathrm{tasks}\\ & y⟼\;\mathrm{subtasks}\;\mathrm{of}\;y \end{array}$$

Let τ be a binary function that tells whether a task is performed or not:

$$\begin{array}{cc} \tau : & T⟶\;\{0,1\}\\ & y⟼\;\tau \left(y\right)=\left\{\begin{array}{c} 1,\mathrm{if}y\mathrm{is}\mathrm{performed}\\ 0,\mathrm{otherwise} \end{array}\right. \end{array}$$

**Task completion constraint.**

We can express that a task is completed if all its subtasks are completed:

(1) $$\tau \left(y\right)=\prod_{z\in sub(y)}\tau \left(z\right)$$

This formula uses the product ∏ to indicate that the task *y* is completed (τ(y)=1) only if all its subtasks τ(z) are completed τ(z)=1. If any sub-task is not completed, the product evaluates to zero, and τ(y) remains zero.

##### Task Dependencies

A task *y* is said to be dependent on another task *x* if *y* cannot start unless *x* was completely performed.

Let dep be a function that associates each task with its dependencies.

$$\begin{array}{cc} dep: & T\to \;PT\;\mathrm{where}\;PT\;\mathrm{is}\;\mathrm{the}\;\mathrm{set}\;\mathrm{of}\;\mathrm{subsets}\;\mathrm{of}\;T\\ & y⟼\;\mathrm{dependencies}\;\mathrm{of}\;y \end{array}$$

**Dependencies constraints**

- **Start time of a task with dependencies** According to the definition, a task cannot be started unless all its dependencies were performed successfully:
  (2) $$start\left(y\right)\geq \max_{z\in \;dep(y)}end\left(z\right)$$
- **Start time of a task with no dependencies**
(3) start(y)≥0
  A task *y* with no dependencies can be independently performed.

#### 3.1.2. Capabilities Definition

Let cap be a function that associates each task with all the required capabilities to perform it.

$$\begin{array}{cc} cap: & T⟶\;PC\;\mathrm{where}\;PC\;\mathrm{is}\;\mathrm{the}\;\mathrm{set}\;\mathrm{of}\;\mathrm{subsets}\;\mathrm{of}\;C\\ & y⟼\;\mathrm{capabilities}\;\mathrm{needed}\;\mathrm{to}\;\mathrm{perform}\;y \end{array}$$

**Capabilities constraints** A task cannot be performed unless all the required capabilities are available:

(4) $$\tau \left(y\right)\leq \prod_{r\in res(y)}isAvailable\left(r\right)\;where$$

$$isAvailable\left(r\right)=\left\{\begin{array}{c} 1,\;\mathrm{if}\;r\;\mathrm{is}\;\mathrm{available}\\ 0,\;\mathrm{otherwise} \end{array}\right.$$

#### 3.1.3. Mission Description

This mission planner needs to receive a clear description of the mission. We describe here a grammar of a language in which this description must be written. The mission planner uses the YAML format corresponding to this language.

The MPL parses this input and produces, as output, a mission plan to be executed. This latter assigns specific tasks to the adequate robots according to the needed capabilities.

**Figure 3:**
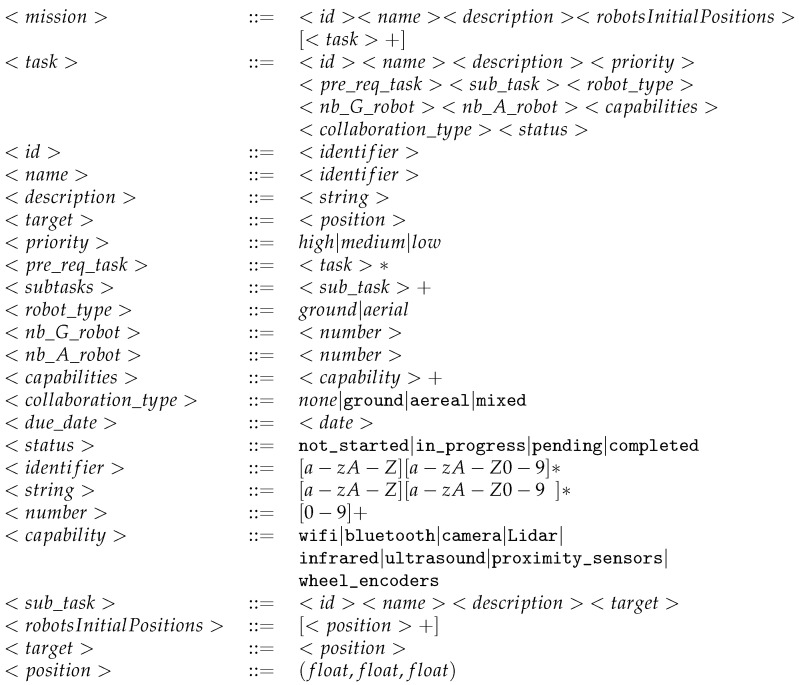
Mission description grammar.

The grammar that we use to describe missions is presented in Figure 3.

#### 3.1.4. Robots Description

**Figure 4:**
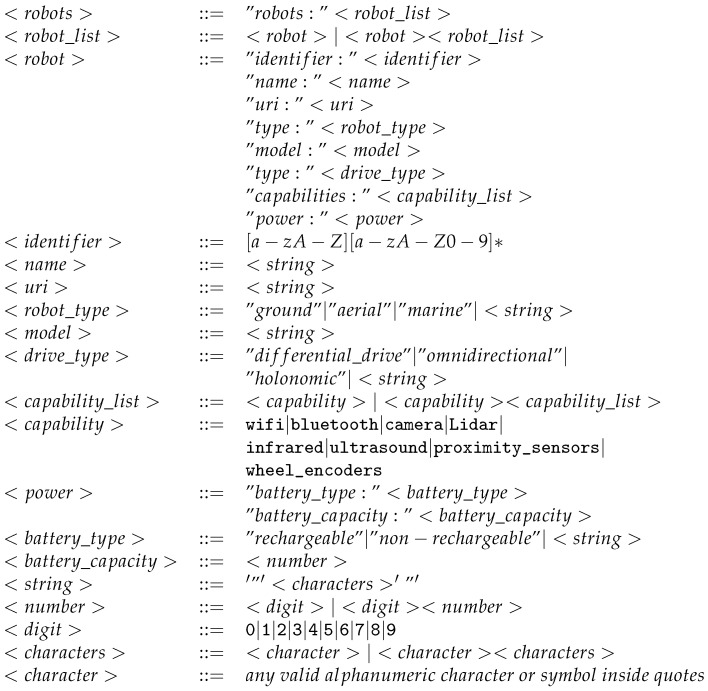
Robot description grammar.

Similarly to the mission description, Figure 4 defines a grammar that describes all available robots in the park, which each robot description should follow. The mission planner uses the YAML format corresponding to this grammar. Examples of robot descriptions can be found in Appendix A; see Figure A3.

The MPL parses this input and produces, as output, the characteristics of all available robots. This enables the selection of the appropriate robots for the mission based on their described capabilities. The MPL then assigns specific tasks to the suitable robots according to the required capabilities.

#### 3.1.5. Mission Plan Builder

Algorithm 1 illustrates the way the mission plan is automatically generated. It parses two input files, namely, the mission description (MD) file and the available pool of robot descriptions (RDs), and then it proceeds with parsing and matching between the requested resources for each task and the provided capabilities of each robot. The output of the process is a mission plan that associates tasks with their needed robots. If we do not find the requested robots, we have a mission failure. Figure A5 shows a Python implementation of Algorithm 1.
**Algorithm 1:** Mission plan builder  **Input**: mission description file (MD) (in YAML format)  **Input**: poolOfRobots description file (RD) (in YAML format)  **Output**: mission plan.

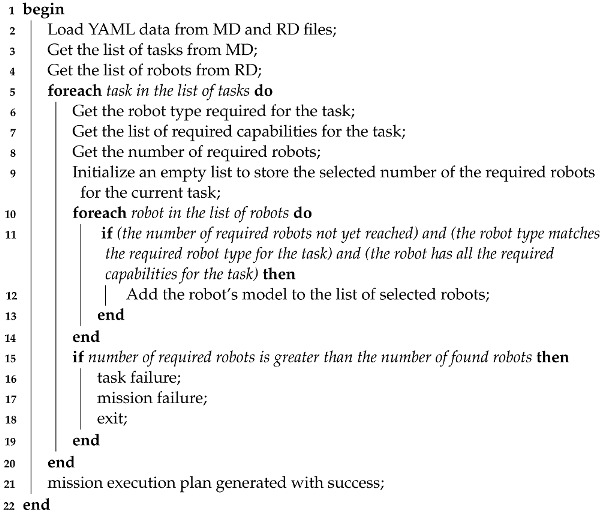


### 3.2. Mission Execution

As mentioned above, a mission consists of a number of tasks that aim to reach some objective.

A mission is to be executed by a possibly heterogeneous set of robots $R_{1},\ldots ,R_{n}$ within an unknown environment. Each robot $R_{i}$ has a driver $Driver_{i}$ that defines its capabilities (the commands that the robot can execute) and allows exchanging information with other robots. It is assumed that all the capabilities needed for the execution of the mission are present among the set of robots.

Each robot $R_{i}$ maintains the two following items that depend on time and whose content changes with the mission:

- Some state, $S_{i}$.
- Some perception of the environment, $P_{i}$.

In order to ensure the common execution of the mission, the individual states are combined into one global state *S*, and the individual perceptions of the environment are combined into one global perception *P*.

The way these combinations are performed changes with the mission. At each instant *t*, a component called Oracle obtains the individual pairs $\left(S_{i}\left(t\right),P_{i}\left(t\right)\right)$ from all the robots through their drivers, $Driver_{i}$, using a module called Worker ($Worker_{i}$), and combines them into the global knowledge K=(S(t),P(t)).

The execution of the mission consists of sending the appropriate commands to the appropriate robots at the appropriate times.

For each robot $R_{i}$, at each instant *t*, a controller $Controller_{i}$ obtains global knowledge K(t)=(S(t),P(t)) from Oracle and makes a decision about the command $C_{i}\left(t\right)$ to be sent to robot $R_{i}$ through its driver $Driver_{i}$.

This implies that each robot makes its own decision locally. Under specific conditions, the controller decides that the mission is finished and stops sending commands to the robot.

**Figure 5:**
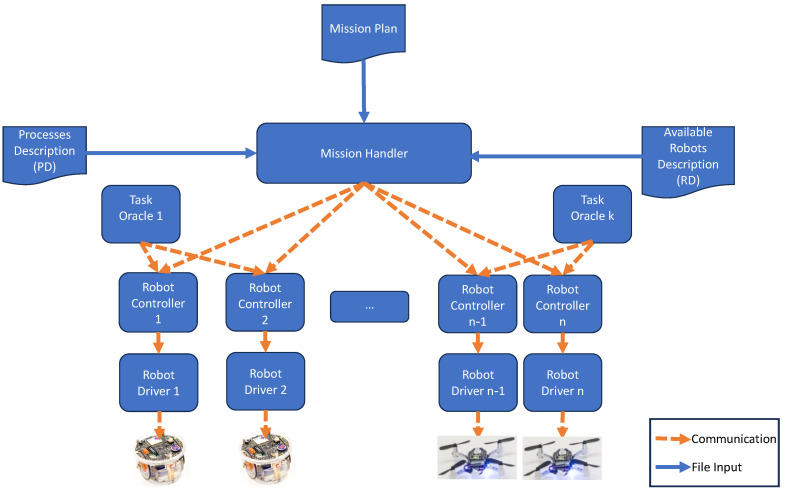
The interactions between the mission−handling modules.

The interactions between all the aforementioned modules (drivers, controllers, oracles) are illustrated in Figure 5.

#### 3.2.1. Mission Handler

**Figure 6:**
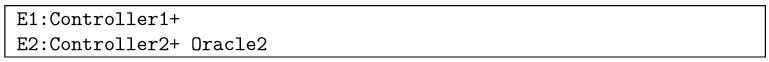
Example of a PD file’s content.

**Figure A1:**
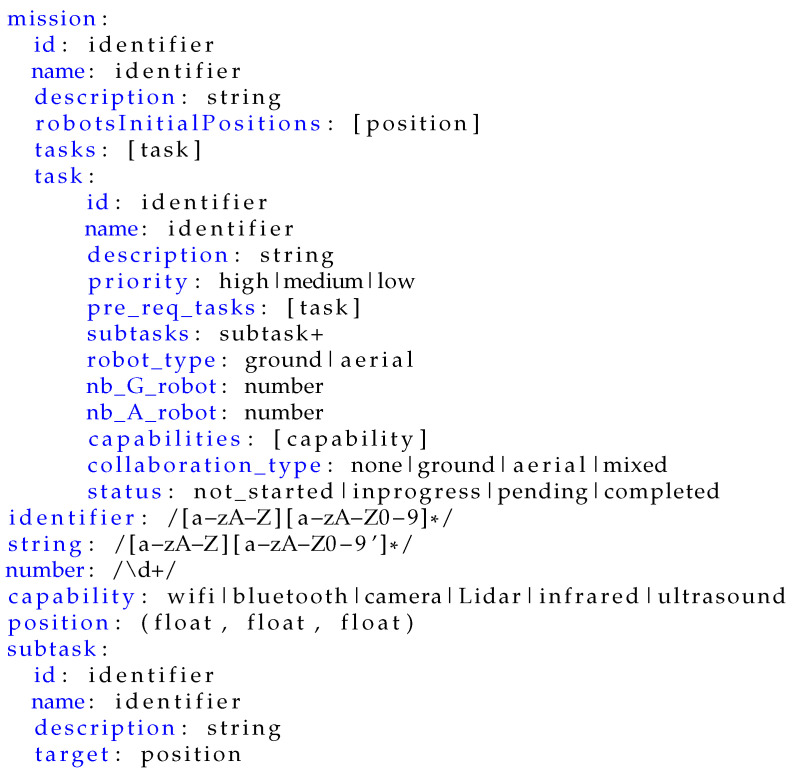
Mission plan grammar in YAML.

The mission handler is responsible for the execution of a mission. It accepts the following inputs:

1. MP: the file containing the mission plan (the format of the file is given in Figure A1 in Appendix A).
2. PD: the file describing the processes needed by different elementary tasks as illustrated in Figure 6.
3. RD: the file describing the pool of available robots. (An example of the file format is given in Figure A3 in Appendix A).

The mission handler extracts the mission elementary tasks from MP along with the associated robots from RD. The execution of an elementary task requires running a set of known processes described in the PD file.

For a given elementary task, the corresponding set of processes includes (at least) robot controllers and robot drivers, as in the case of task E1 in Figure 6. Some tasks may require an oracle process that provides controllers with necessary information about the environment and the group of robots, as demonstrated by task E2 in Figure 6.

#### 3.2.2. Robot Driver

**Figure 7:**
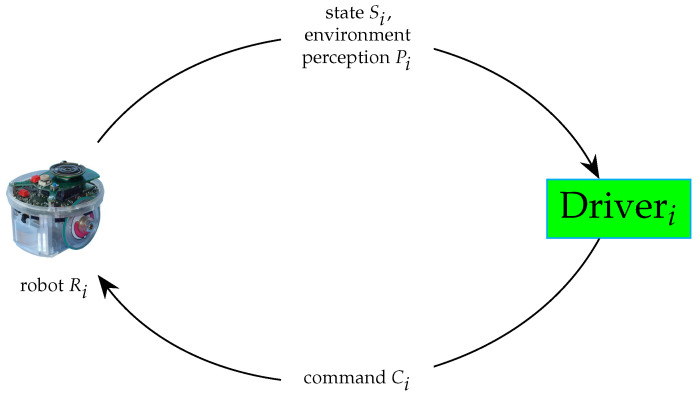
Robot driver.

A robot driver is responsible for exposing the communication interface with a single robot. So, it obtains information from the robot regarding its state and its observable environment and can send commands to the robot, as depicted in Figure 7.

Examples of state information include the position coordinates of the robot and its battery level. However, an obstacle encountered or a camera image are examples of environmental information. Common commands to ground robots include moving forward or turning left/right.

The driver is platform-dependent and can expose an interface to a real robot or a simulation environment. In both cases, it hides the physical details of the robot.

#### 3.2.3. Robot Controller

A robot controller is responsible for the execution of an elementary task by a single robot. It is also responsible for notifying the mission handler about the termination of tasks. A multi-robot task requires as many controllers as participating robots. A robot controller receives the elementary task to be executed, along with the initial robot configuration, from the mission handler. To fulfill its responsibilities, the controller sends the appropriate commands to the robot through its driver.

**Figure 8:**
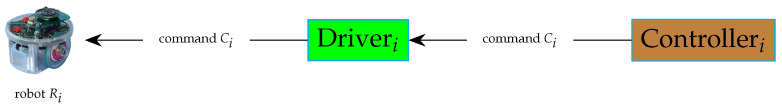
Interaction between a controller and a driver: case of a single-command task.

In the case of a single-operation elementary task, the controller sends a single command to the robot and finishes. This is illustrated in Figure 8.

In the case of a general elementary task, more than one command will be needed. As we assume a discrete time, with each tick of the clock $t_{j}$ the controller sends a new command to the robot through the driver. The sent command is based on the following:

1. The current global state $S(t_{j})$ of the group of robots;
2. The current global perception of the environment $P(t_{j})$;
3. The task completion state expressed in Equation (1).

For each robot, a worker software module was added to the framework for synchronization purposes. All the workers send the states and the environment perceptions associated with the robots, respectively, to the oracle at the same clock tick.

**Figure 9:**
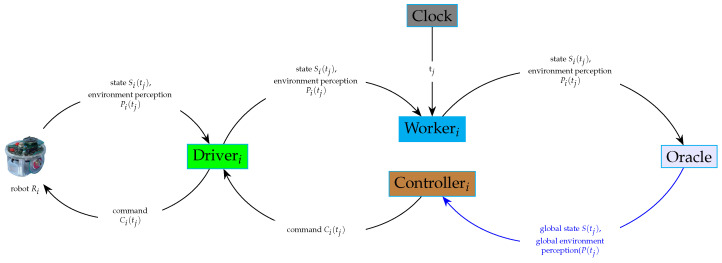
Interaction between a controller, a driver, and an oracle in the case of a multiple-commands task.

This is illustrated in Figure 9.

Hence, the controller will have a state whose evolution can be described by an automaton. For a task that involves a formation composed of robots of the same type, the robot controller checks—at each instance—whether the target was reached. If not, it uses the automaton to calculate the next controller state and the next command to be sent to the robot. An example of such an automaton is given in the case study in Section 4.

**Figure 10:**
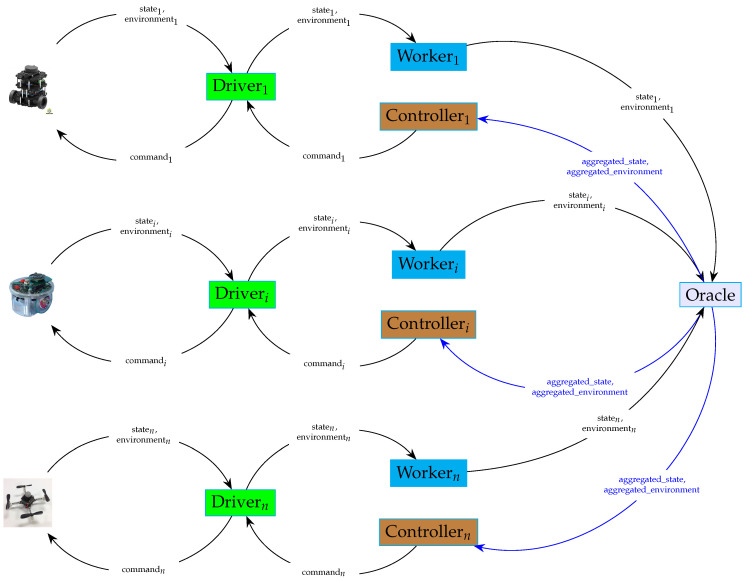
Interactions of software modules during the execution of a multi-robot task (state_*i*_: state *S_i_*(*t_j_*), environmenti: environment perception *P_i_*(*t_j_*), aggregated_state: global state *S*(*t_j_*), aggregated_environment: global environment perception: *P*(*t_j_*)).

Figure 10 depicts the interactions between different modules during the execution of a multi-robot task. It highlights the role of the oracle in synchronizing the decisions made by the controllers and performed by the robots. It does so by collecting information about the current states and discovered environments from different robots.

#### 3.2.4. Task Oracle

The task oracle is responsible for providing the global state and environment information about the involved robots needed by the controllers. Note here that a multi-robot task needs, at most, one oracle.

The oracle will then need to gather state and environment information from individual robots. For each robot, a worker process will be responsible for obtaining information from the driver and feeding it to the oracle. This communication pattern is represented in Figure 9.

Depending on the task, the oracle will aggregate the inputs in order to produce the global state and the discovered environmental information. This occurs at each tick of the clock.

For a task that involves a formation composed of robots of the same type, the individual state (including at least the position and the movement angles) at a time $t_{j}$ of a given robot *i* could be stated as follows:

(5) $$S_{i}\left(t_{j}\right)=\left(x_{i}\left(t_{j}\right),y_{i}\left(t_{j}\right),z_{i}\left(t_{j}\right),yaw_{i}\left(t_{j}\right),pitch_{i}\left(t_{j}\right),roll_{i}\left(t_{j}\right)\right)$$

Based on the set of individual states gathered, the oracle calculates the global state (including at least the position of the formation mass center and the movement angles averages) at the same time tick $t_{j}$, as follows:

(6) $$S\left(t_{j}\right)=\left(\frac{\sum_{i=1}^{n}x_{i}\left(t_{j}\right)}{n},\frac{\sum_{i=1}^{n}y_{i}\left(t_{j}\right)}{n},\frac{\sum_{i=1}^{n}z_{i}\left(t_{j}\right)}{n},\frac{\sum_{i=1}^{n}yaw_{i}\left(t_{j}\right)}{n},\frac{\sum_{i=1}^{n}pitch_{i}\left(t_{j}\right)}{n},\frac{\sum_{i=1}^{n}roll_{i}\left(t_{j}\right)}{n}\right)$$

In the case of the existence of obstacles, we note *K* as the number of sides of the robot where an obstacle can be detected. The environmental perception by an individual robot *i* (including boolean values that represent the sides where an obstacle is detected) at time $t_{j}$ can be expressed as follows:

(7) $$P_{i}\left(t_{j}\right)={\left({\mathrm{obstacle}}_{i,k}\left(t_{j}\right)\right)}_{1\leq k\leq K}$$

where ${\mathrm{obstacle}}_{i,k}\left(t_{j}\right)$ is *True* if the robot *i* detects an obstacle on side *k* at time $t_{j}$, and *False* otherwise.

The global environment perception could then be calculated at time $t_{j}$ as the disjunction of the boolean values denoting the obstacles detected by different robots:

(8) $$P\left(t_{j}\right)={\left(\overset{n}{\underset{i=1}{⋁}}{\mathrm{obstacle}}_{i,k}\left(t_{j}\right)\right)}_{1\leq k\leq K}$$

In the Case Study section, we will show an instantiation of the aforementioned equations using a mission example.

#### 3.2.5. Processes Communication

The mission handler, robot controllers, robot drivers, and task oracle are implemented as ROS 2 nodes [21]. They communicate using ROS 2 topics and services, which facilitates the creation of loosely coupled software modules.

#### 3.2.6. Platform-Independence

As elaborated above, the controllers, the workers, and the oracle are platform-independent. In other words, the same modules can be reused for robots from different vendors having the same capabilities.

The robot driver is the only platform-dependent module, as it encapsulates specific low-level details about robots.

## 4. Mission Case Study

The goal of this case study is to demonstrate how the previously defined framework can be applied in simulating a multi-robot mission, without loss of generality regarding the framework’s broader applicability. The case study involves the following key aspects:

- A heterogeneous set of robots;
- An unknown environment;
- An obstacle along the path.

The considered mission is an exploration mission, where the robots are asked to move to a given target position in an unknown environment, take photos, send them to some destination, and return to their initial positions. It involves two ground robots equipped with cameras and an areal robot. All the robots have obstacle-detection capabilities.

In the sequel, we detail the different steps implemented through the proposed framework to accomplish the mission.

### 4.1. Mission Planning

**Figure A2:**
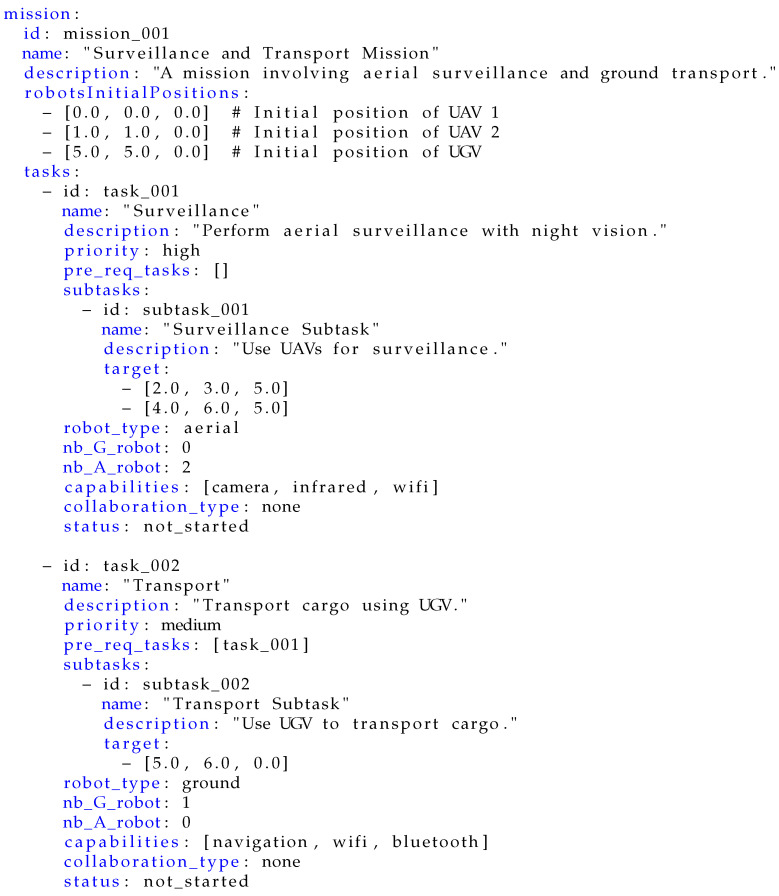
Mission description (MD).

**Figure A3:**
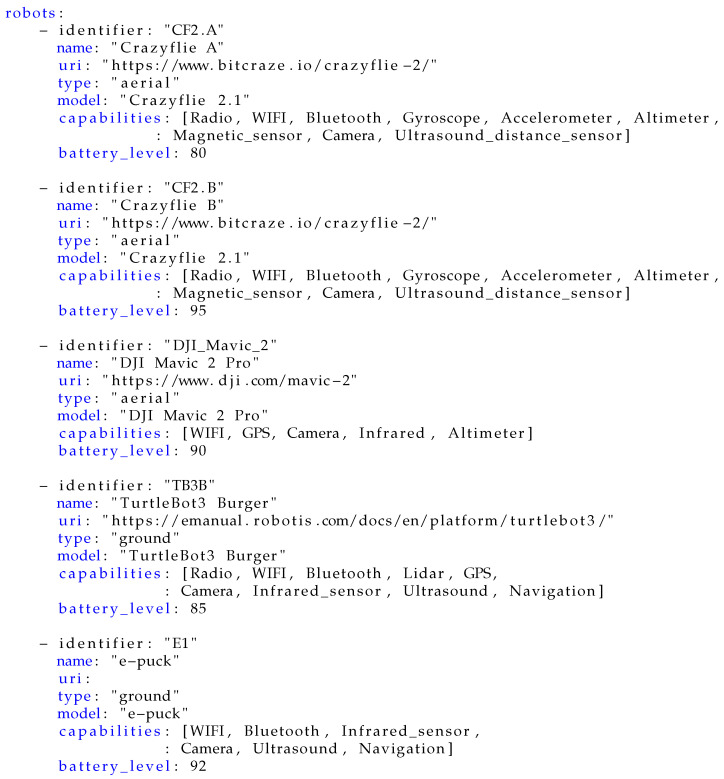
Robot pool specification.

**Figure A5:**
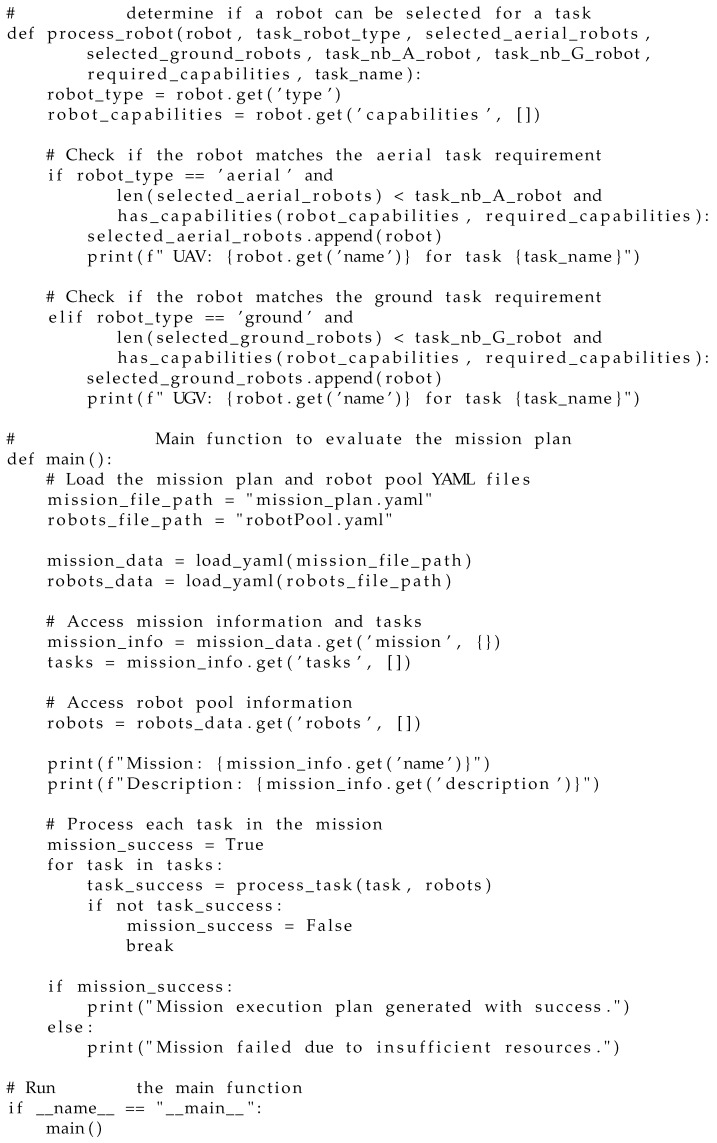
Mission planner process—part 2.

The inputs to the mission planner, represented in Figure 1 include the MD file describing the mission and the RD file describing the available robots. Samples of these files are depicted in Figure A2 and Figure A3, respectively.

The mission plan is then automatically generated, as illustrated in Table 1, and given as input to the mission execution part, which presents the following subsections.

### 4.2. Mission Execution

#### 4.2.1. Input Files

Table 1 shows the contents of the three input files (MD, RD, and PD) used in the case study. Recall that MD, RD, and PD are defined in Section 3.2.1.

According to the MD and RD files, we have three robots, named EP1, EP2, and CF1. In this case study, EP1 and EP2 are Epuck robots [22] while CF1 is a Crazyflie robot [23,24]. EP1 and EP2 are two-wheeled robots, whereas CF1 is a quadrotor (nano-drone). All the robots are equipped with sensors used to detect obstacles. Three elementary tasks are listed in the file MD:

1. The first elementary task consists of moving the ground robots from their initial positions to a given target position while keeping a rigid formation and avoiding possible encountered obstacles. Meanwhile, the areal robot follows their trajectory.
2. The second elementary task involves the three robots taking and sending photos from their positions, reached after executing the first task, to a specified FTP server.
3. Finally, the three robots move back to their initial positions under the same conditions as the first elementary task.

In the sequel, we will focus on the first elementary task and the processes involved in its execution. We will refer to EP1, EP2, and CF1 as $R_{1}$, $R_{2}$, and $R_{3}$, respectively.

#### 4.2.2. The Mission Handler

The mission handler parses the file MD and extracts the first elementary task GoToTarget that has to be executed by the three robots $R_{i}$ (i∈{1,3}). Then, the mission handler launches the appropriate drivers listed in the file RD. By examining the PD file, it initiates one ground_goto_controller process for each robot and a goto_oracle process.

#### 4.2.3. The Task Oracle: goto_oracle

As time is discrete, the state of an individual robot $R_{i}$ (i∈{1,3]}) at an instant $t_{j}$ is defined by its position and heading. By instantiating Equation (5), we obtain the following expression for the state of an individual ground robot:

(9) $$S_{i}\left(t_{j}\right)=\left(x_{i}\left(t_{j}\right),y_{i}\left(t_{j}\right),{\mathrm{heading}}_{i}\left(t_{j}\right)\right),\mathrm{where}i\in \left\{1,2\right\}$$

The state of the areal robot $R_{3}$ at an instant $t_{j}$ is defined by an additional third dimension, denoted as $z_{i}\left(t_{j}\right)$. Based on the states of the individual robot, the oracle calculates the global state. By instantiating Equation (6), we obtain the following expression for the following global state:

(10) $$S\left(t_{j}\right)=\left(\frac{\sum_{i=1}^{2}x_{i}\left(t_{j}\right)}{2},\frac{\sum_{i=1}^{2}y_{i}\left(t_{j}\right)}{2},\frac{\sum_{i=1}^{2}{\mathrm{heading}}_{i}\left(t_{j}\right)}{2}\right)$$

The function of $R_{3}$ will basically do the same thing, but since we have only one aerial robot, the aggregated state will be the same as the individual one.

For a ground robot, obstacles can be detected on three sides: ahead, left, and right. Thus, the environment of a robot $R_{i}$ (i∈{1,3]}) is given at time $t_{j}$ by three Boolean values that indicate whether the robot encounters an obstacle ahead, to the left, and/or to the right, respectively. By instantiating Equation (5) for the ground (respectively, aerial) robots, we obtain the following expression for the environment discovered by an individual robot at time $t_{j}$ noted $P_{i}^{g}$ (respectively, $P_{i}^{a}$):

(11) $$P_{i}^{g}\left(t_{j}\right)=\left(\mathrm{obstacle}\_{\mathrm{ahead}}_{i}\left(t_{j}\right),\mathrm{obstacle}\_{\mathrm{left}}_{i}\left(t_{j}\right),\mathrm{obstacle}\_{\mathrm{right}}_{i}\left(t_{j}\right)\right)$$

(12) $$P_{i}^{a}\left(t_{j}\right)=\left(\mathrm{obstacle}\_{\mathrm{ahead}}_{i}\left(t_{j}\right),\mathrm{obstacle}\_{\mathrm{left}}_{i}\left(t_{j}\right),\mathrm{obstacle}\_{\mathrm{right}}_{i}\left(t_{j}\right)\right)$$

The aggregated environment is given at time $t_{j}$ by the disjunction of each of the Boolean values for the two ground robots. By instantiating Equation (8), we obtain the following expression for the global environment discovered by the ground robots at time $t_{j}$:

(13) $$P^{g}\left(t_{j}\right)=\left(\overset{2}{\underset{i=1}{⋁}}\mathrm{obstacle}\_{\mathrm{ahead}}_{i}\left(t_{j}\right),\overset{2}{\underset{i=1}{⋁}}\mathrm{obstacle}\_{\mathrm{left}}_{i}\left(t_{j}\right),\overset{2}{\underset{i=1}{⋁}}\mathrm{obstacle}\_{\mathrm{right}}_{i}\left(t_{j}\right)\right)$$

A similar equation is used to calculate $P^{a}\left(t_{j}\right)$.

The result of the aggregation is then communicated to the robot controllers at each instant $t_{j}$.

#### 4.2.4. The Robot Controller

ground_goto_controller

**Figure 11:**
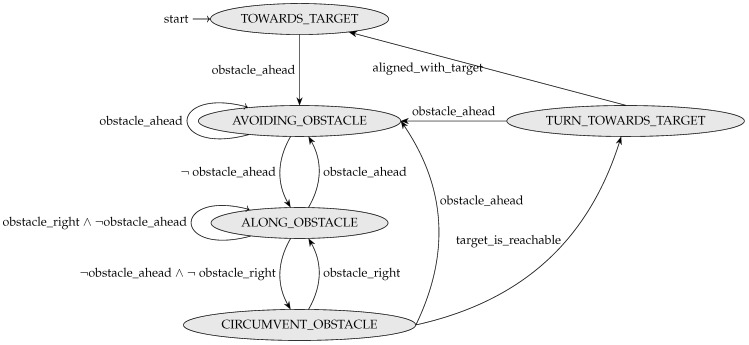
Automaton used by the controller ground_goto_controller.

At each instance $t_{j}$, the ground_goto_controller process checks whether the target was reached. If not, it uses the automaton shown in Figure 11 to calculate the next controller state. The next command to be sent to the robot is then determined as a function of the controller state according to Table 2.

aerial_goto_controller

**Figure 12:**
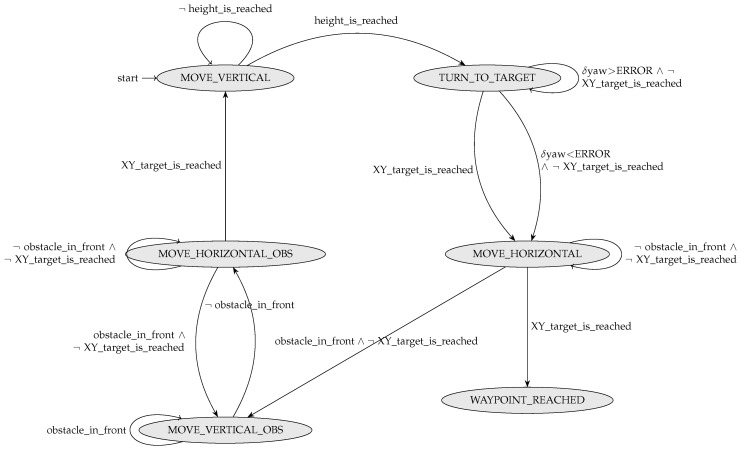
Automaton used by the controller aerial_goto_controller.

Similar to the ground robot controller, at each instant $t_{j}$, the aerial_goto_controller process checks whether the target was reached. If not, it uses the automaton shown in Figure 12 to calculate the next controller state.

Recall that the aerial robot follows the ground robots in the air, trying not to lose their trace while avoiding obstacles and selecting optimal flight paths. Hence, the target for the aerial robot is influenced by the current positions of the ground robots.

### 4.3. A Simulation Example

We simulate the case study using the Webots simulator [25] in the ROS 2 environment.

Webots is an open-source and multi-platform desktop application used to simulate robots. It provides a complete development environment to model, program, and simulate robots.

**Figure 13:**
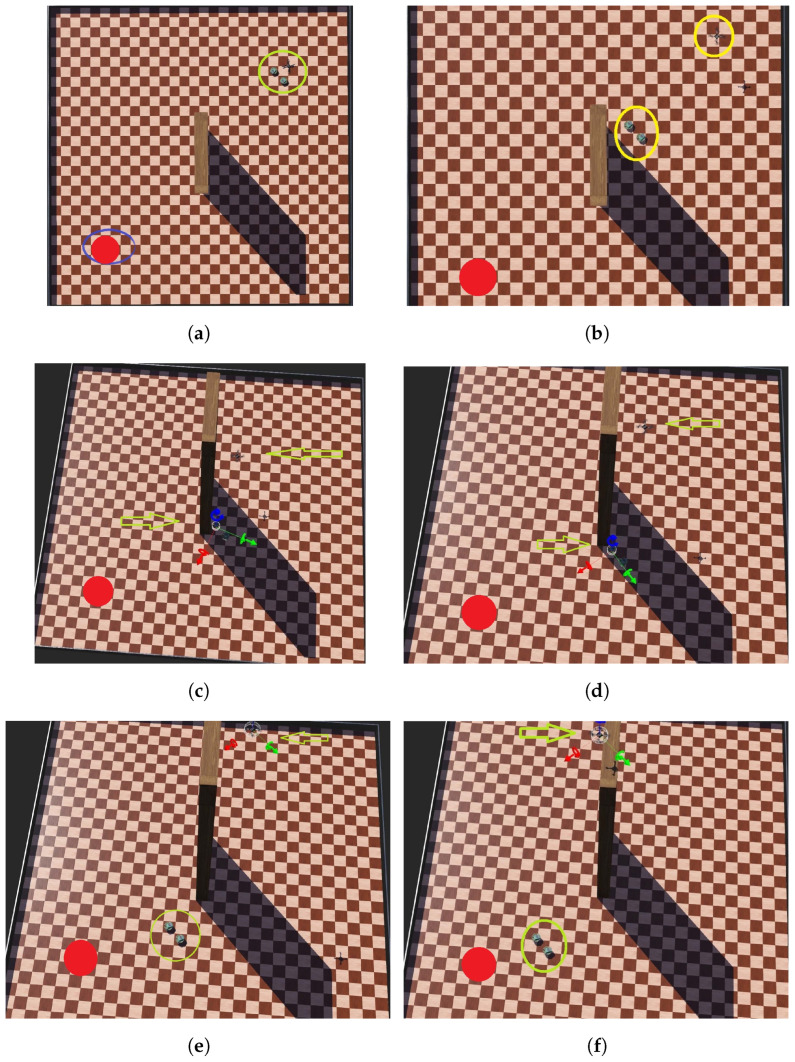
Robots move to a target point while avoiding an obstacle (part 1). (**a**) Initial configuration. (**b**) The robots move toward the target. (**c**) The first e-puck robot detects an obstacle. (**d**) UGVs change direction and move. UAV detects an obstacle. (**e**) UGVs move toward the target. UAV ascends in order to avoid the obstacle. (**f**) UAV flies over the obstacle.

The initial and target positions for each robot are predefined throughout the entire mission simulation, and the robots maintain their original formation. Figure 13a illustrates the start of the simulation, where the initial positions are marked in yellow and the target positions in red.

At the beginning of the mission, all three robots move toward the target point (as depicted in Figure 13b). When the first e-puck robot detects an obstacle (shown in Figure 13c), the ground robots adjust their path to avoid it by moving parallel to the obstacle. After reaching the end of the obstacle, the robots maneuver around it to continue toward the other side (as shown in Figure 13d). Once the obstacle is cleared, the robots resume their movement toward the target (Figure 13e).

**Figure 14:**
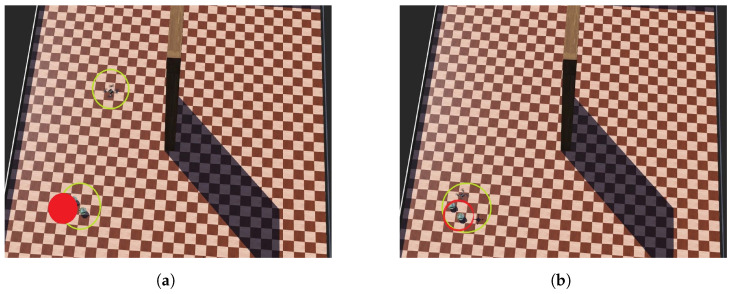
Robots move to a target point while avoiding an obstacle (part 2). (**a**) UAV tracks UGVs. (**b**) The robots reach the target position.

Meanwhile, the aerial robot, upon detecting an obstacle (as seen in Figure 13d), ascends until it reaches the top of the obstacle (Figure 13e). It then flies over the obstacle (Figure 13f) and continues tracking the ground robots (Figure 14a) until they all arrive at the target position (Figure 14b).

## 5. Conclusions and Future Work

This paper introduced a framework for mission planning and execution in heterogeneous multi-robot systems, focusing on the integration of UAVs and UGVs to handle mission scenarios that require both 2D and 3D formations. The framework’s decentralized control strategies are shown to be effective in optimizing task allocation, maintaining formation stability, and enabling robust collaboration. The framework emphasizes modularity and ease of integration for essential mission components such as drivers, controllers, workers, aggregators, and oracles. This makes the framework scalable and adaptable to a range of mission types, from simple to complex.

The presented case study served as a proof of concept, demonstrating the framework’s foundational capabilities in a simulated environment. The case study illustrated a sample implementation of the framework’s components in an unstructured environment with a heterogeneous set of robots. It showcases a part of the framework’s potential for handling highly dynamic missions.

One of the key contributions of this paper involves the potential for expanding the framework into a more robust tool, ultimately supporting mission planning and execution via an intuitive graphical interface. In real-world applications, this framework would enable users to design and execute missions involving hybrid groups of UAVs and UGVs. To realize this potential, further development is needed to address several challenges associated with deploying the framework on real robots, including system latency and environmental uncertainty.

Future work will also explore the integration of advanced AI techniques, such as machine learning and reinforcement learning, to enhance the framework’s decision-making and real-time adaptability. These methods would allow the system to learn from previous missions, optimizing task allocation and formation control in increasingly complex environments.

In summary, while the current work lays the groundwork for mission planning and execution in multi-robot systems, further investigation may be necessary to fully demonstrate its effectiveness in real-world scenarios.

## Figures and Tables

**Table 1:** Input data used in the case study.

MD	GoToTarget(50,100,0): EP1(0,3,0) EP2(4,0,0) CF1(3,4,0)
	TakeAndSendPhoto(sftp://server.com): EP1() EP2() CF1()
	GoToTarget(0,0,0): EP1() EP2() CF1()
RD	EP1: Epukdriver…
	EP2: Epukdriver…
	CF1: Crazyfliedriver…
PD	GoToTarget(a,b,c): ground_goto_controller+ aerial_goto_controller+ goto_oracle
	TakeAndSendPhoto(server_uri): ground_send_photo_controller+

**Table 2:** The commands issued by the controller ground_goto_controller as functions of its state.

**State**	**Command**	**Explanation**
TOWARDS_TARGET AVOIDING_OBSTACLE	forward turn_left	Move toward the target in a straight line When in front of an obstacle, turn left until it becomes possible to move forward without hitting the obstacle
ALONG_OBSTACLE CIRCUMVENT_OBSTACLE	forward turn_right	Move forward “in parallel” with the obstacle When reaching the end of the obstacle, turn around its extremity in order to move to the other side
TURN_TOWARDS_TARGET	turn_left OR turn_right	Once o the other side (behind an obstacle), turn toward the target

**Figure A4:**
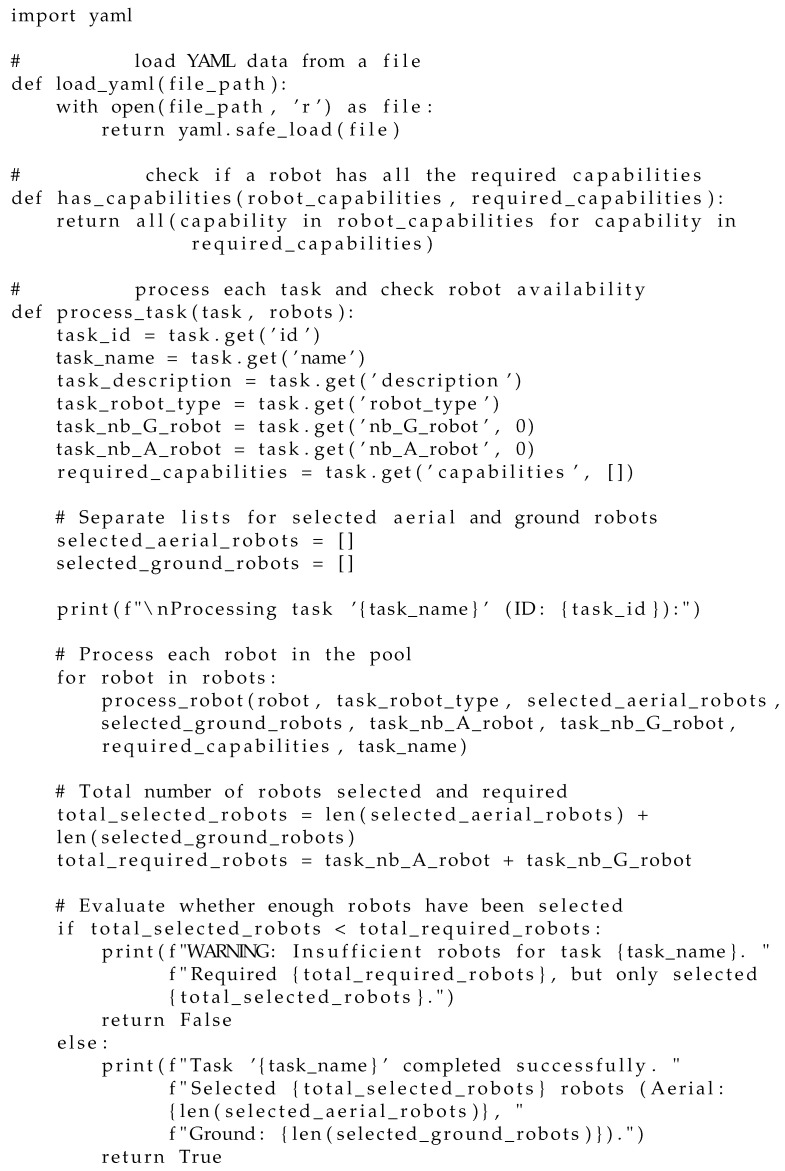
Mission planner process—part 1.

## Data Availability

No new data were created or analyzed in this study. Data sharing is not applicable to this article.
